# Exploring self-determined solutions to service and system challenges to promote social and emotional wellbeing in Aboriginal and Torres Strait Islander people: a qualitative study

**DOI:** 10.3389/fpubh.2023.1206371

**Published:** 2023-09-22

**Authors:** Anna P. Dawson, Eugene Warrior, Odette Pearson, Mark A. Boyd, Judith Dwyer, Kim Morey, Tina Brodie, Kurt Towers, Sonia Waters, Cynthia Avila, Courtney Hammond, Katherine J. Lake, ‘Uncle’ Frank Lampard, ‘Uncle’ Frank Wanganeen, Olive Bennell, Darrien Bromley, Toni Shearing, Nathan Rigney, Schania Czygan, Nikki Clinch, Andrea Pitson, Alex Brown, Natasha J. Howard

**Affiliations:** ^1^Wardliparingga Aboriginal Health Equity, South Australian Health and Research Institute, Adelaide, SA, Australia; ^2^Adelaide Medical School, Faculty of Health and Medical Sciences, The University of Adelaide, Adelaide, SA, Australia; ^3^Division of Medicine, Northern Adelaide Local Health Network, Adelaide, SA, Australia; ^4^College of Medicine and Public Health, Flinders University, Adelaide, SA, Australia; ^5^Aboriginal Services, AnglicareSA, Adelaide, SA, Australia; ^6^Aboriginal Health, Sonder, Adelaide, SA, Australia; ^7^Indigenous Health Equity, Centre for Health Equity, Melbourne School of Population and Global Health, The University of Melbourne, Carlton, VIC, Australia; ^8^Executive Office, Kaurna Elder and Aboriginal Community Representative, Adelaide, SA, Australia; ^9^Executive Office, Nunga Mi:Minars Inc., Adelaide, SA, Australia; ^10^Executive Office, InCompro Inc., Adelaide, SA, Australia; ^11^Aboriginal Health Promotion, Wellbeing SA, Adelaide, SA, Australia; ^12^Statewide Operations, South Australian Department for Corrections, Adelaide, SA, Australia; ^13^Aboriginal Education Directorate, South Australian Department for Education, Adelaide, SA, Australia; ^14^Indigenous Genomics, Telethon Kids Institute, Adelaide, South Australia, Australia; ^15^National Centre for Indigenous Genomics, Australian National University, Canberra, ACT, Australia

**Keywords:** Aboriginal and Torres Strait Islander peoples, social and emotional wellbeing, indigenous methodologies, yarning circles, qualitative, social determinants of health, health and social services

## Abstract

**Introduction:**

Many Aboriginal and Torres Strait Islander people living on Kaurna Country in northern Adelaide experience adverse health and social circumstances. The *Taingiwilta Pirku Kawantila* study sought to understand challenges facing Aboriginal and Torres Strait Islander communities and identify solutions for the health and social service system to promote social and emotional wellbeing.

**Methods:**

This qualitative study applied Indigenous methodologies undertaken with Aboriginal and Torres Strait Islander governance and leadership. A respected local Aboriginal person engaged with Aboriginal and Torres Strait Islander community members and service providers through semi-structured interviews and yarning circles that explored community needs and challenges, service gaps, access barriers, success stories, proposed strategies to address service and system challenges, and principles and values for service design. A content analysis identified the breadth of challenges in addition to describing key targets to empower and connect communities and optimize health and social services to strengthen individual and collective social and emotional wellbeing.

**Results:**

Eighty-three participants contributed to interviews and yarning circles including 17 Aboriginal community members, 38 Aboriginal and Torres Strait Islander service providers, and 28 non-Indigenous service providers. They expressed the need for codesigned, strengths-based, accessible and flexible services delivered by Aboriginal and Torres Strait Islander workers with lived experience employed in organisations with Aboriginal and Torres Strait Islander leadership and governance. Community hubs and cultural events in addition to one-stop-shop service centres and pre-crisis mental health, drug and alcohol and homelessness services were among many strategies identified.

**Conclusion:**

Holistic approaches to the promotion of social and emotional wellbeing are critical. Aboriginal and Torres Strait Islander people are calling for places in the community to connect and practice culture. They seek culturally safe systems that enable equitable access to and navigation of health and social services. Aboriginal and Torres Strait Islander workforce leading engagement with clients is seen to safeguard against judgement and discrimination, rebuild community trust in the service system and promote streamlined access to crucial services.

## Introduction

Colonisation in Australia created a myriad of complex challenges impacting contemporary Aboriginal and Torres Strait Islander peoples (1). Australia was colonised with racist practices that accomplished dispossession, displacement and marginalisation (2) enabled by discriminatory government policies that mandated the forced removal of children and exclusion from health, employment and education opportunities (3). The targeted and multigenerational actions of the colonial project continue to manifest in the lives of Aboriginal and Torres Strait Islander people (1, 4) in the form of systemic racism (5) and inequitable income, employment, education and psychosocial distress (6) alongside barriers to access for both health care (7) and community services (8). Access to services is fraught due to unequal access to financial resources, transport challenges (7), lack of cultural safety, communication difficulties, referral challenges, absence of Aboriginal and Torres Strait Islander support services (9), stigmatisation and fear of discrimination (10). Such barriers to crucial health care undoubtedly contributes to unacceptable and inequitable health and wellbeing outcomes (4).

Racism damages physical and mental health (11, 12) and threatens service access (13). Social disadvantage is concurrent with poor general and mental health (5, 14), social and emotional wellbeing (15) and chronic disease (16). Without targeted approaches that tackle social risks and unmet social needs related to the social determinants of health (17, 18), holistic health outcomes in Aboriginal and Torres Strait Islander peoples will never be realised. Yet leaders highlight the importance of protective cultural elements and warn that approaches targeting the social determinants of health alone are ‘insufficient to understand contributions to our health and wellbeing’ (p.S12) (19). Access to culturally safe services is imperative and innovative solutions to service system challenges are long overdue.

Aboriginal and Torres Strait Islander conceptualisations of health are holistic, encompassing the physical, social, emotional and cultural wellbeing of individuals and their communities (20). Aboriginal and Torres Strait Islander people are calling for greater consideration of cultural factors in policy and program design (19, 21). The cultural determinants of Aboriginal and Torres Strait Islander health include connection to Country, Indigenous knowledges, language, family, kinship and community, cultural expression and continuity, and self-determination and leadership (22). Gee and colleagues’ (23) model of Aboriginal social and emotional wellbeing articulates a holistic understanding of health and highlights the centrality of connection to culture, spirituality and Country and the importance of relationships with family, kin and community. It provides nuance in conceptualizing how these factors are shaped and influenced by historical, political and social determinants. Hence both social and cultural determinants of health, in the context of political and historical factors, are proposed as key levers for strengthening social and emotional wellbeing.

Government attempts at combating the disadvantage carried by Aboriginal and Torres Strait Islander people in Australia have failed to consider protective cultural factors for more than a decade of Closing the Gap policy initiatives (24). Culture, language, land rights and social and emotional wellbeing were only recently included as priorities when targets were codesigned with Aboriginal and Torres Strait Islander people (25). Indigenous-led codesigned approaches are vital in the context of a violent colonial history since they represent power sharing and rebuild trust (26). There are numerous examples of effective codesign approaches with Aboriginal (27–29) and First Nations peoples internationally (30, 31) from which we can draw.

Action on the social and cultural determinants of health presents many challenges across service settings and community environments. This is particularly true in Aboriginal and Torres Strait Islander communities that are forced to face multi-level and multi-generational health and social challenges. The northern region of Adelaide in South Australia, on the traditional lands of the Kaurna people,^1^ is one of the most disadvantaged areas of Australia being consistently rated in the highest quartile of the Australian Bureau of Statistics’ Index of Relative Socioeconomic Disadvantage (32). People living here experience disproportionate unemployment and welfare reliance, less education, and more frequent mental health, and drug and alcohol challenges (33). The 2021 Census found 18.1% (n = 7,690) of South Australia’s Aboriginal and Torres Strait Islander population resided in this catchment (34). Within this region, Aboriginal and Torres Strait Islander communities are diverse and many are growing in strength despite the impacts of colonisation, yet they experience double the rate of suicide, 5.6 times the rate of drug-related hospitalisations, and up to 9.2 times the rate of alcohol-related deaths than the non-Indigenous population in the region (35). Like many other regions, the health and social service system is fragmented and uncoordinated (8) undoubtedly compounding the challenges facing Aboriginal and Torres Strait Islander people.

Considering the plethora of challenges impacting Aboriginal and Torres Strait Islander communities in northern Adelaide, the aim of the study was to respectfully engage Aboriginal and Torres Strait Islander people and service providers on Kaurna Country to gather local perspectives informed by lived experiences related to community needs, service challenges and perceived solutions to promote social and emotional wellbeing. These self-determined solutions were collected to inform efforts for health and social service system reform necessary to achieve *Taingiwilta Pirku Kawantila*; which, in Kaurna language, is ‘Strong Community in the North’.^2^

## Materials and methods

### Study design

This qualitative study represents the first stage in *Taingiwilta Pirku Kawantila*, a 5-year mixed-method program of research that seeks to examine whether codesigned, tailored, multi-level strategies to optimise and coordinate the health and social service system can meet the needs of Aboriginal and Torres Strait Islander peoples and strengthen social and emotional wellbeing. An Indigenous methodological approach guides this research program from conception through to data collection, analysis and dissemination. The research was designed specifically to privilege local Indigenous voices (36) and knowledges (37) and to ‘acknowledge community members as co-designers, co-implementers and co-knowledge translators of strengths-based research and outcomes’ (21) (p.72). The research is being undertaken by the Wardliparingga^3^ Aboriginal Health Equity research group at the South Australian Health and Medical Research Institute and was designed in response to research priorities identified by Aboriginal communities across South Australia (38).

The study investigators include two senior Aboriginal and Torres Strait Islander chief investigators and four non-Indigenous chief investigators. There are three Aboriginal and one non-Indigenous associate investigators who are embedded in the service sector as leaders in service provision for Aboriginal and Torres Strait Islander people. A respected local Aboriginal person in the role of Senior Engagement and Knowledge Broker connects the research team (comprising three Aboriginal and two non-Indigenous research personnel) with the Aboriginal and Torres Strait Islander community and service providers in northern Adelaide. The study’s Aboriginal Governance Panel comprise Aboriginal Elders and workforce living and/or working in northern Adelaide. The Panel oversees research processes and provides cultural and contextual guidance in relation to community engagement processes, data collection methods, interpretation of data and meaningful translation of findings to benefit community. Local ethical principles for Aboriginal health research are followed throughout all research processes (39). The study is being undertaken with ethical approvals from the Aboriginal Health Research Ethics Committee of South Australia (04–20-885) and the Northern Adelaide Local Health Network (HREC 14241) in addition to clearances from numerous institutional ethics committees.

### Study region and setting

*Taingiwilta Pirku Kawantila* is undertaken on the traditional lands of the Kaurna people in northern Adelaide, South Australia. The city of Adelaide was established by colonisers in the early 1840s in a place known to Kaurna people as *Tarntanyangga* which means ‘place of the red kangaroo’ (40). The Kaurna people, who never ceded sovereignty of their Lands, were forced off their Country by settlers during this time (41). This experience of dispossession and displacement was echoed for many Aboriginal and Torres Strait Islander nations across Australia. As a result, living across suburbs of northern Adelaide there are local Kaurna people along with Aboriginal and Torres Strait Islander people from other ancestral Country across South Australia and other states and territories of Australia. The project region is the catchment of the Northern Adelaide Local Health Network. This region was selected as the focus of this research due to the considerable number of Aboriginal and Torres Strait Islander residents (34) and a high degree of need in relation to both socioeconomic disadvantage (32) and challenging health and social outcomes (35).

### Participant recruitment and data collection

The Senior Engagement and Knowledge Broker recruited Aboriginal and Torres Strait Islander community members and service providers to semi-structured interviews (with one participant) and yarning circles (with a group of participants). Service providers were employees of health and social service organisations who provided programs and services to Aboriginal and Torres Strait Islander people across the northern region of Adelaide. The Indigenous research method of yarning, described as an ‘Indigenous cultural form of conversation’ (p.37), was used with Aboriginal and Torres Strait Islander community members and service providers to build relationships (i.e., social yarning) and explore research questions (i.e., semi-structured research topic yarning) (42). Proposed yarning prompts were reviewed and revised by the Aboriginal Governance Panel prior to data collection. Participants were provided with written information about the study and an opportunity to ask questions prior to providing informed consent. They were also informed that a Distress Protocol was in place which outlined the supports available (e.g., counselling) if distress was experienced during the process of yarning about community needs and challenges. Cultural protocols were followed prior to yarning including acknowledging Country, introductions to connect to family and place, and pausing for a Minute of Silence to show respect to those that have passed away (43). Yarning was facilitated by the Senior Engagement and Knowledge Broker (EW) in all but one instance where an interview undertaken at a domestic violence service was facilitated by a female non-Indigenous researcher (AD) and a female Aboriginal researcher (TB) since it was inappropriate for a male researcher to be on site. AD was present to take notes during 20 of the 30 interviews and yarning circles. Yarning prompts were designed so that participants could first share their lived experiences and observations of community needs and challenges, service gaps and access barriers before identifying success stories, proposing solutions for reform, and identifying principles and values to guide service provision. Interviews were undertaken face to face in all but one instance where a participant with whom the research team had an existing relationship requested to participate via videoconferencing. The Senior Engagement and Knowledge Broker made contact in the days following participation to undertake a welfare check and thank interviewees for their time. The Distress Protocol was not applied in any instance. Participants shared their own lived experiences and also recounted stories of clients or fellow community members, known as ‘shadowed data’ (44). Interviews and yarning circles were digitally recorded with consent and transcribed verbatim in all but two instances where institutional ethical clearances deemed that only notes could be taken for use in analysis. Data collection continued until the research team felt confident that a broad range of perspectives from Aboriginal and Torres Strait Islander community members and service providers and non-Indigenous service providers were represented.

### Data analysis

Data from all interviews and yarning circles were analysed together. Transcribed text and written notes were analysed in NVivo software (QSR International Pty Ltd) by EW and AD. We applied the coding methods of Saldana (45) and content analysis approach of Graneheim and Lundman (46) in identifying sections of transcribed text that represented meaning units, ascribing codes, and grouping codes into categories and sub-categories. The first five transcripts were inductively coded (45), yielding extensive codes. We then reviewed and discussed the codes before developing and applying a framework for the dataset that included multilevel community needs and challenges, challenges with services and systems, and proposed solutions for a strengthened service system (including actions; principles, values, and ways of working; and barriers and enablers of implementation). Structural coding (45) followed, with inductive coding of remaining interviews and yarning circles within the framework. EW and AD met regularly to review and revise codes, categories, and sub-categories to best represent the perspectives of participants and facilitate rapid translation of findings. Following careful coding and consideration of the dataset’s manifest content, a thematic analysis was undertaken to identify common perspectives shared by respondents that could guide community leaders, health service administrators and policy makers in identifying key targets for system, service, and practice reform. As a process of collective member checking, the research team presented codes and emerging themes to the project’s Aboriginal Governance Panel across four quarterly meetings and invited their input in interpreting the findings. This process promoted rigour and ensured the findings adequately reflected the lived experiences of Aboriginal and Torres Strait Islander communities in northern Adelaide.

## Results

We collected data with Aboriginal community members via four interviews and four yarning circles, and with service providers via eight interviews and 14 yarning circles. The sample, described in Table 1, comprised 1 Torres Strait Islander and 54 Aboriginal participants, including 17 Aboriginal community members (Elders, men, women, youth, and a survivor of the Stolen Generations^4^) and 38 Aboriginal and Torres Strait Islander people who brought to yarning discussions both their lived experience as members of the northern Adelaide community and their professional perspectives as members of the Aboriginal health and social services workforce. There were also 28 non-Indigenous participants who were employed in government, non-government, and Aboriginal community controlled^5^ health and social service organisations in northern Adelaide. Participants were predominantly female, including around 60% of Aboriginal community members and Aboriginal and Torres Strait Islander service providers, and more than 80% of non-Indigenous service providers. Yarning circles with service providers commonly included both Aboriginal and Torres Strait Islander and non-Indigenous colleagues. The mean (range) duration of interviews and yarning circles was 73 min (31–148 min) with Aboriginal community members and 72 min (40–153 min) with service providers.

**Table 1:** Description of Participants.

	Aboriginal community members	Aboriginal and Torres Strait Islander service providers	Non-Indigenous service providers	Total
Men	7	13	4	24
Women	10	25	24	59
Total	17	38	28	83
**Employment Sector**				
Health		9	4	13
Education		9		9
Legal		4	1	5
Family Services		3	4	7
Disability		5	4	9
Housing			9	9
Homelessness		2	2	4
Police		2	2	4
Drug and Alcohol		1		1
Domestic Violence		1		1
Mental Health		1		1
Community Services			2	2
Human Services		1		1
Total		38	28	66
**Organisation**				
Government		20	14	34
Non-government Organisation		2	4	6
Aboriginal Community Controlled Organisation		16	10	26
Total		38	28	66

### Community challenges

A plethora of community challenges were shared, with narratives of poverty and financial concerns saturated throughout participant recounts in addition to lack of transport, housing and rental challenges, homelessness and overcrowding.

*You put poverty into the equation and parents that think I can’t afford $20 next week. I can’t afford the petrol money and the medical appointment and the doctor’s bill. And I can’t afford that psychology appointment.*

*(Homelessness and youth services sector, Yarning circle)*

Experiences of racism, discrimination, ignorance, systems of control and oppression and use of deficit narratives were frequently described. Other harms of colonisation such as experiences of childhood removal as part of the Stolen Generations, and disconnection from culture and cultural identity were also shared. Intergenerational contact with the criminal justice and child protection systems were described, perpetuated by an absence of cultural connection and identity leading individuals to seek unhealthy connections and substance use for belonging and escape. Family and relationship challenges were frequently described in relation to domestic violence, vulnerable young people, and children under Guardianship Orders.^6^ Other relationship challenges included lateral violence, community politics, Elder abuse and social isolation.

*Participant: Generally, 60% of our tenants would be 55 and over, about 60% of them would be living on their own, so we have quite an aged cohort.*

*Interviewer: Yeah. And solo living.*

*Participant Solo living, yeah and a lot of social isolation. It’s a big issue.*

*(Non-Indigenous service provider, Housing sector)*

Unsurprisingly, health concerns were extensive with mental health challenges (e.g., experiences of grief and loss, trauma, shame and self-harming) and mental health diagnoses (e.g., anxiety; depression; schizophrenia; bipolar; attention deficit hyperactivity disorder), alcohol and other drug challenges, disability and chronic diseases described. As discussed in a yarning circle with Aboriginal staff supporting children and families:

*Participant: A lot of mental health. That’s the huge issue here.*

*Participant: Wow. We hear – that’s what we hear every day normally, don’t we?*

*Participant: Every day. Mental health, yeah.*

*Participant: So we’re working with - - -*

*Participant: System’s shithouse.*

*Participant: We’re working with parents that are bipolar, depression, anxiety, drug and alcohol - - -*

*Participant: Domestic violence.*

*Participant: Domestic violence. Yep. But mental health. You’re right [Name] hit it on the head.*

*(Aboriginal service providers, Education sector)*

### Barriers to service access: organisation-level and system-level

**Figure 1:**
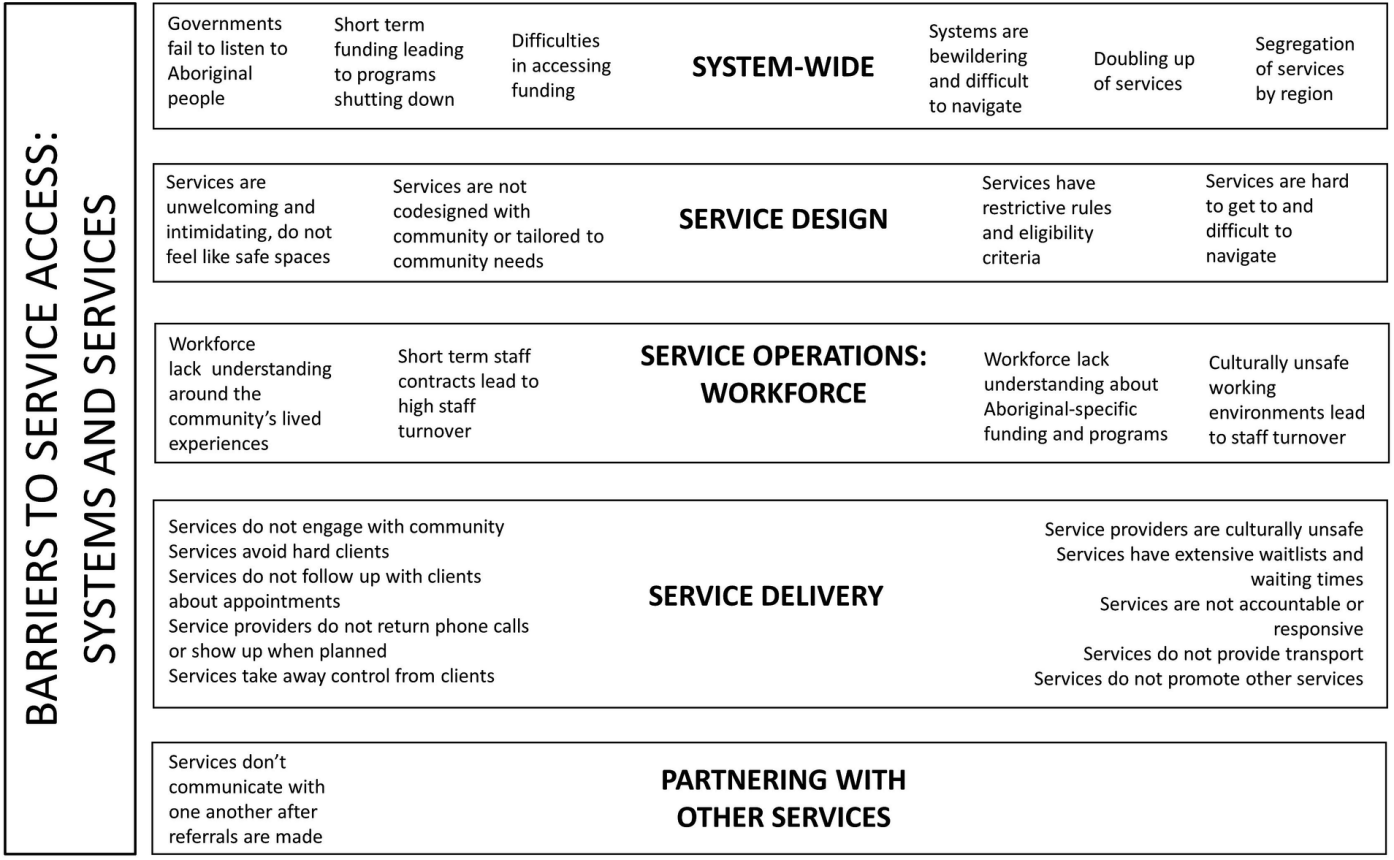
System and service level barriers to health and social services’ access.

There were a range of access barriers identified at the level of health and social services in relation to how services work with community, how services are designed, how services are delivered, and how services work with partner organisations. Workforce challenges were also frequently identified. In addition, there were numerous system-level barriers to service access described by participants. These barriers are presented in Figure 1.

### Barriers to service access: personal

**Figure 2:**
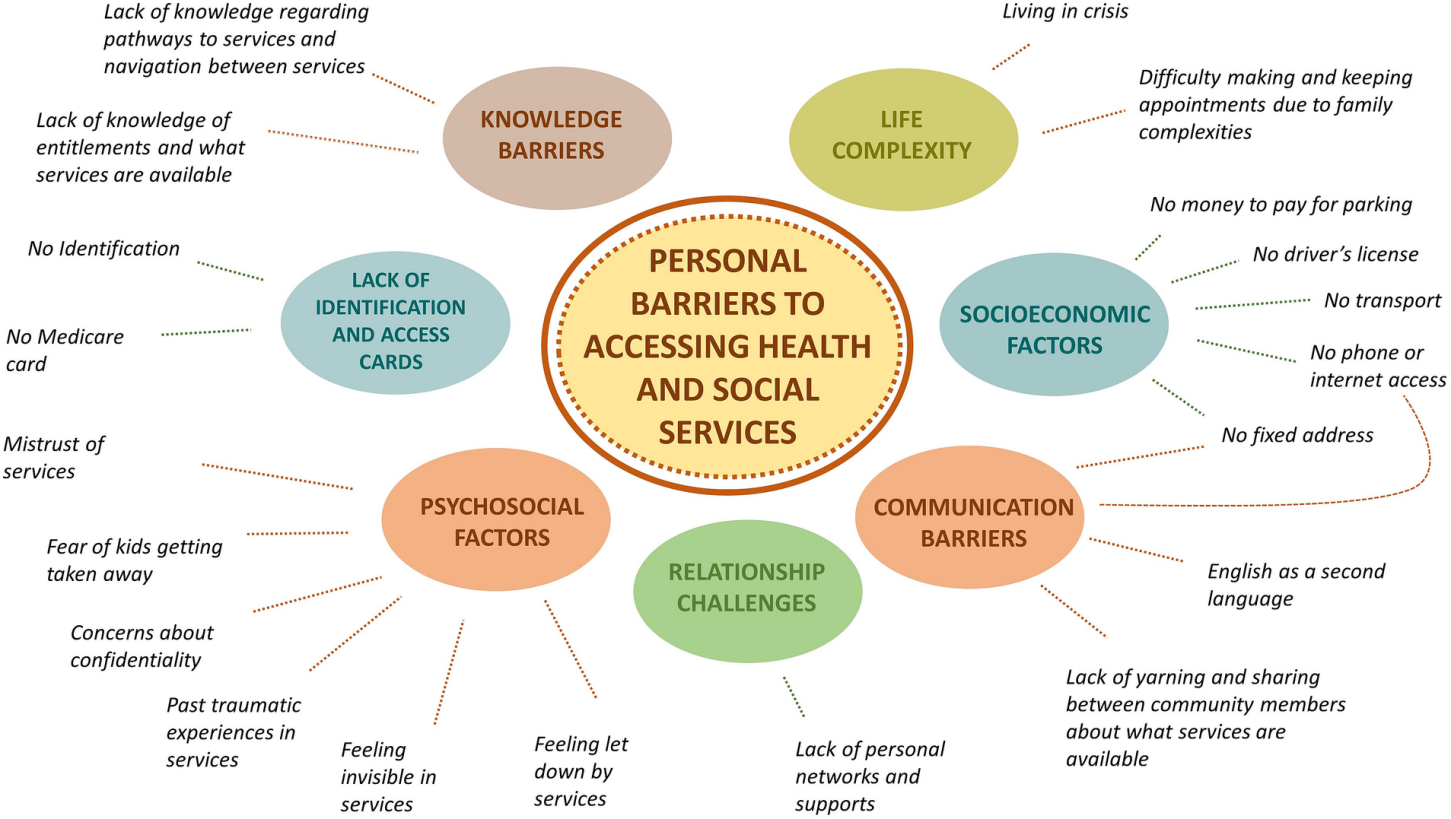
Personal barriers to accessing health and social services.

Participants described extensive personal barriers to service access faced by Aboriginal and Torres Strait Islander people in northern Adelaide. These barriers are depicted in Figure 2 and include psychosocial factors, socioeconomic challenges, life complexities, knowledge barriers, relationship challenges, communication challenges, and lack of personal identification and access cards. A Survivor of the Stolen Generations spoke of avoiding services and systems for fear of her children being taken away. Service providers spoke of challenges for Aboriginal and Torres Strait Islander people who come to Adelaide from remote areas of the state and interstate, particularly in relation to social isolation, homelessness, language and communication challenges and in relation to receiving culturally safe and responsive services tailored to their needs.

### General challenges with services

Service challenges were identified at the level of governance, operations, service design, service delivery and inter-agency partnerships. Lack of Aboriginal decision making, particularly by local Kaurna people, was identified as a governance concern. A range of cultural safety challenges in service operations were voiced including institutionalised and systemic racism, interpersonal racism between colleagues, and lack of culturally responsive service providers. Additional operational problems included funding restrictions, insufficient workspaces to run programs, and challenges with auditing and creating reports from client record systems. There were widespread workforce challenges including lack of staff training and understanding, unclear roles and responsibilities, lack of support, short term contracts, and experiences of abuse from clients. Stories of culturally incompetent workforce were shared in accounts of workers wrongly judging Aboriginal clients’ mental health behaviours as inebriation due to stereotypes. For Aboriginal and Torres Strait Islander workers there were specific challenges such as being subject to tokenism, high expectations from clients, the stress of living and working in community and a lack of Aboriginal and Torres Strait Islander workers across services.

*And I think that there’s a huge barrier in the expectations are so much higher for us, that we have to be 100% all the time, working for community, but also living in community, being held accountable by community, there’s not - I don’t see fairness in – there’s not a safe space, I guess, for those discussions to be had with Indigenous and non-Indigenous people in the workplace.*

*(Aboriginal service provider, Legal sector)*

In relation to service design, the challenges centred around a failure to tailor services to the needs of clients. Respondents described services that are out of touch with community needs, that do not listen to and engage with community through true community protocols and that have rigid systems that set Aboriginal and Torres Strait Islander people up to fail.

*We don’t listen to the voice; we don't listen to what the individual wants, and they slip through the cracks. But the cracks are the cracks that we've created; I don’t believe people slip through cracks. I believe that we haven't listened to what they needed.*

*(Aboriginal service provider, Homelessness sector)*

Participants also spoke of services that failed to create welcoming spaces for Aboriginal and Torres Strait Islander clients:

*And but, you know, I see especially Sisters and Aunties and they go, and they go, you know, ‘Doctor wasn't listening. Doctor doesn't understand. Nothing in the rooms to show, you know, that this is this is a safe space, culturally safe space for Aboriginal people’. And of course, that translates into if there are no aesthetics or no visuals around that, then how will that define the treatment or drive the treatment of you as an Aboriginal, as an Aboriginal person?*

*(Aboriginal community member, Survivor of the Stolen Generations)*

In relation to service delivery, respondents spoke of lack of client centred models of care, unresponsive services, judgement from services, punitive rather than restorative approaches, insufficient time for clients and workforce to connect, and use of deficit language:

*I don't even talk about service-resistant. That's the one thing, when a service says to me, “Oh, you know, we've been trying to help blah, and she’s service-resistant.” And I said, “Well, no, she's not service-resistant, it's your service that is not responding to her need.”*

*(Aboriginal service provider, Homelessness sector)*

Lack of continuity in services was another major challenge, as was services not dealing with underlying or core issues, only consequences and crisis management:

*I think something I’ve also found in my very limited experience of working with Aboriginal people and Aboriginal organisations is that we felt that we were forever chasing the consequences opposed to the issue. Like, we weren’t actually addressing the core issue of what is going on in that person’s life.*

*(Non-Indigenous service provider, Community services sector)*

A range of shortfalls in service provider behaviours were described. Examples were provided where service providers judged clients or told clients what to do rather than providing support for clients to make their own decisions. Instances were described where workers failed to ask clients about their needs, to explain what was going to happen with a procedure, or to seek permission to have another service provider present.

*So, you know, it's sort of like or they do turn up and then it's like permission isn't asked, you know, for somebody to accompany them.*

*(Aboriginal community member, Survivor of the Stolen Generations)*

In relation to working with other services, the predominant challenges related to lack of partnerships, communication, trust, information sharing and case management meetings. There were challenges raised in gaining consents to share information and issues with ineffective referral processes.

### Gaps in services

There were several services that were identified as under-resourced, struggling to meet the needs of the community or insufficient across the region. The gaps identified by community members and service providers included a lack of Aboriginal-specific services; lack of mental health services, particularly in relation to counsellors and pre-crisis supports; gaps in drug and alcohol services including sobering up centres and rehabilitation facilities; lack of housing, homelessness and crisis accommodation services; gaps in the correctional system in relation to diversionary programs, prison release programs and lack of Aboriginal and Torres Strait Islander staff; lack of youth programs and services; lack of mentorship and role model programs; lack of family violence accommodation for mothers with older male children; lack of dental services; and lack of social programs across the region.

*Interviewer: So, what are the big gaps out there in services from your perspectives that you think need to be addressed?*

*Participant: Mental health and keeping in contact with clients within mental health services. There are people I find in hospital situations with, say, anyone from the community that goes in with a mental health issue, it doesn’t get followed up, they get discharged, they’re back out in the community with no help, no nothing. So that is a big one with mental health, yeah.*

*(Aboriginal community member, Youth yarning circle)*

### Systems-level challenges

Respondents spoke of challenging systems-level factors in relation to the way services are funded and the requirements to report against key performance indicators (KPIs). Specifically, they spoke of services chasing funding and KPIs rather than client outcomes, and of a system that created competition between services:

*So, I think it’s also because there’s so many tenders in the industry now and you’re competing for the same cohort. You’re not working for the same cohort, you’re competing against another service, so that wall of mistrust is so high, it’s like “I’m not going to share with you, because you’re going to encroach on my clients”. Actually no, they’re our clients. Why aren’t we working together to better this?*

*(Aboriginal service provider, Homelessness sector)*

Participants also identified short term program funding and withdrawal of funding for innovative programs as key challenges. They highlighted that, due to time pressures, the health and social service system deals only with client symptoms and fails to deal with underlying issues. They felt that in some instances services were not being held accountable, however over-regulation of Aboriginal money was also identified as a concern and reforms were seen to be laborious. Lack of cultural safety training at university training programs was seen to threaten cultural safety across the system.

### Principles, values and ways of working

**Figure 3:**
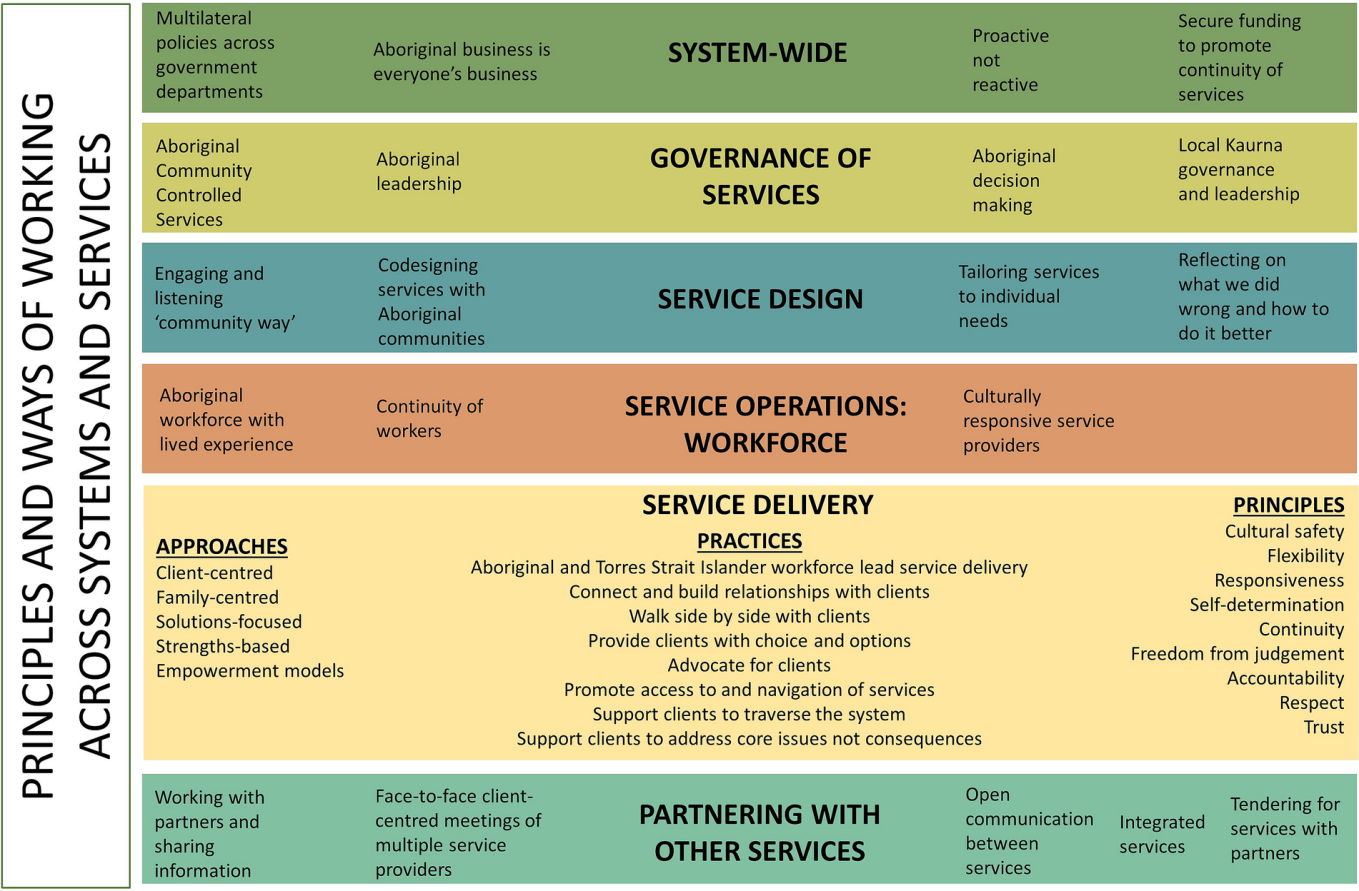
Recommended principles and ways of working across systems and services in northern Adelaide.

The principles, values and ways of working proposed by participants across the service system, in organisational governance and leadership, operations, service design in service delivery, and in working in partnership with other services are highlighted in Figure 3.

Client-centred and family-centred approaches were highlighted as important in service delivery alongside solutions-focused and strengths-based practices, empowerment models, and Aboriginal and Torres Strait Islander workforce leading engagement with clients. In relation to specific practices, workforce participants spoke of connecting and building relationships with clients, providing clients with choice and options, advocating for clients, promoting access to and navigation of services, walking side by side, having navigator roles to support clients to traverse the system and, importantly, supporting clients to deal with underlying core issues not only consequences.

*Participant: So, in terms of a value and a principle, then quick response times is what we need, we need things to be - - -*

*Participant: It needs to be more responsive than it is, especially if you've got a client sitting there at risk and like where she lived basically was here, and we were here. And he was looking for how do we keep her safe, if you're not going to respond and get her into a safe, secure location, then we basically are used as a barrier, and it's not acceptable, it really isn't.*

*Participant: So, it's to be responsive, and then also to have Aboriginal representation in all of the organisations visible.*

*(Health sector service providers, Yarning circle)*

The key principles identified included cultural safety, flexibility, responsiveness, self-determination, continuity, freedom from judgement, accountability, respect and trust. As a worker in family services highlighted:

*You know, when you think about all of the services that are available and all of the gaps, the gap is the right to be self-determined to make those changes. It is a big thing, Aboriginal self-determination and self-reliance. But that right is not given, it is controlled. (Family services sector, Yarning circle)*

### Enablers and barriers to implementing Aboriginal and Torres Strait Islander ways of working.

Workforce factors were seen to be the primary enablers of implementing the identified principles, values and ways of working. These centred around Aboriginal and Torres Strait Islander workforce with lived experience and connections who get results alongside supportive non-Indigenous workers with *“a really good heart for Nunga*^7^ *people” (Aboriginal service provider, Mental Health sector).*

*What I'm finding is helping me get past some of the barriers is - I've got to use my community relationships. So, I've kind of gone around the back door. So, I did that with [name of organisation] the other day. I went straight to the person in charge because I know them personally and that got me 24 hours turnover for referrals but that's how important is our relationships with community, other community members, and just trying to navigate past the crap …*

*(Aboriginal service provider, Health sector)*

Supportive chief executive officers and leadership were seen to be important as were effective partnerships and alliances secured by legal agreements. The identified barriers included a lack of Aboriginal and Torres Strait Islander staff, lack of cultural safety (e.g., lack of mandated cultural safety training, lack of cultural knowledge), inflexible mainstream organisations that have barriers to Aboriginal and Torres Strait Islander ways of working, inadequate consultation and codesign, insufficient funding and time constraints.

### Self-determined solutions to address challenges and promote social and emotional wellbeing

**Figure 4:**
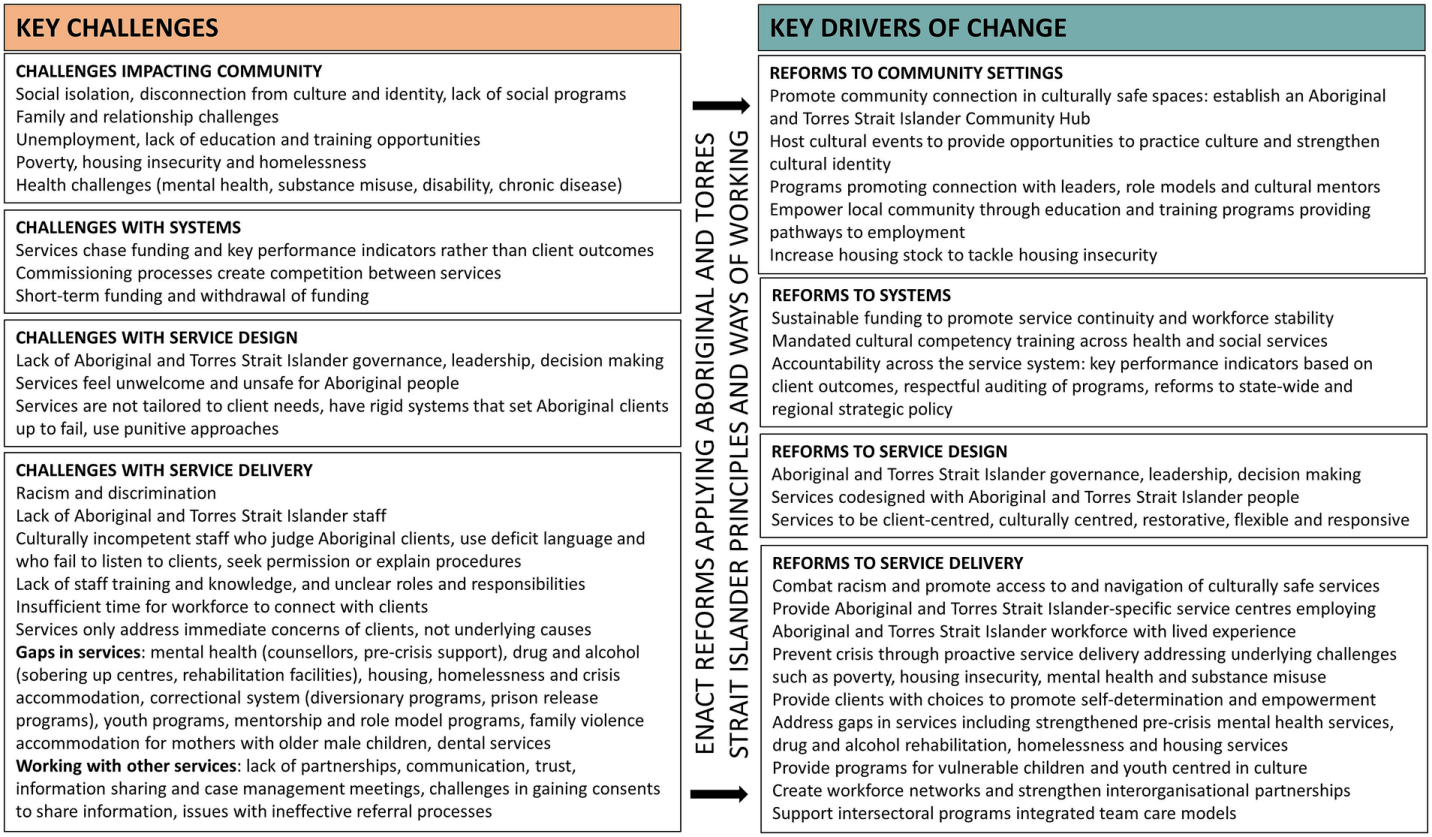
Key challenges and drivers of change across community settings and the health and social service system.

There were numerous key messages threaded across the dataset in considering local perspectives on community needs and service challenges, the principles and values to guide service delivery, and proposed strategies for system reform. Figure 4 provides a summary of the challenges described by Aboriginal and Torres Strait Islander people and service providers in northern Adelaide and highlights proposed strategies and key drivers of change targeting community settings and the health and social service system.

At the community level we heard that infrastructure to promote community connection in culturally safe spaces and programs that strengthen cultural practices and identity are integral to the promotion of social and emotional wellbeing in Aboriginal and Torres Strait Islander people living in northern Adelaide. This was reflected in consistent calls for an Aboriginal community hub, community and cultural events, and for community leaders and cultural mentors.

*That's right. One community engagement. Yep. Everyone within the community knows what's going on. It's a meeting point, to a place where you can sit around and do whatever, other services coming in. Yeah, perfect.*

*(Aboriginal community member, Survivor of the Stolen Generations)*

Strategies to promote empowerment and self-determination were repeatedly voiced, articulated through Aboriginal and Torres Strait Islander governance, leadership and decision making across services; through education and training opportunities; through programs linking community with local leaders, role models and mentors; and through service delivery models that provide clients with information, choices, and decision-making opportunities. A raft of strategies that promoted access to and navigation of culturally safe services were proposed in response to racism and both client and service-level access barriers. These included Aboriginal and Torres Strait Islander-specific service centres, employing Aboriginal and Torres Strait Islander workforce with lived experience who are connected to community, who provide ‘tough love’ by setting expectations and demanding accountability from clients, and who can advocate for clients and promote navigation and coordination across a complex system.

*Don’t pussy-foot around with your families because I think that’s where you gain the trust and respect because you’re going in there, you’re telling them up front, what’s the best, the worst outcome possible. But if you do come work with me and we do this, this is going to be a better outcome for you. That’s the way. And I know [Name] is the same and I know [Name] is the same. She’s out telling her parents, what for. But they’re young parents and they’re still learning, they’re still growing.*

*(Aboriginal service provider, Education sector)*

Mandated training to build the cultural capacity of non-Indigenous staff was considered vital since experiences of racism, judgement and disrespect lead Aboriginal and Torres Strait Islander community members to feelings of shame and mistrust and to avoidance of services and systems. Strengthened interorganisational partnerships and workforce networks were considered necessary to promote client navigation of health and social services, and intersectoral activities such as case management meetings and programs that brought agencies together to deliver integrated care were often highlighted among success stories. We consistently heard calls for strengthened pre-crisis services in mental health and drug and alcohol rehabilitation as these challenges are risk factors for domestic violence, vulnerable children and over-representation in the justice system.

We heard that, fundamentally, compounding problems must be prevented through proactive service delivery addressing underlying causal factors such as poverty, housing insecurity, substance challenges and mental health concerns since the system often responds only after clients are entrenched in crisis. Participants spoke of programs for vulnerable children centred in culture. There were consistent calls for strengthened programs for children and youth across the region such as On Country programs, mentoring programs with Elders, and youth mental health services. The importance of Aboriginal and Torres Strait Islander workforce to guide and mentor children removed from families and placed under Guardianship Orders was emphasised, as was the importance of welcoming and culturally safe accommodation.

Respondents highlighted that when developing programs, services must engage with and deeply listen to Aboriginal and Torres Strait Islander people. Thus, codesigning services with the Aboriginal community was considered essential as was continuity in services that were client-centred, culturally centred, flexible, and responsive. The need for sustainable funding to promote service continuity and workforce stability was voiced frequently. Finally, accountability across the service system was considered paramount since some services “*avoid hard clients*” or “*manage them out of the service*.” Accountability measures for governments and services were framed through the development of co-designed state-wide and regional strategies and policy, through carefully constructed key performance indicators centred on client outcomes, and through respectful auditing of services in receipt of Aboriginal money.

*But I think it comes down to the government saying, “If we give you money to provide a service to Aboriginal people, you will be culturally audited. You will be ethically audited. You will be accountable to make sure that your best practice framework is culturally safe, is culturally fit, is culturally sensitive.” And just making people accountable for taking funds that are to represent and improve the social and emotional wellbeing of Aboriginal people and being held accountable to it.*

*(Non-Indigenous service provider, Community services sector)*

Notably, there were several instances where participants shared a sense of despondency, that while challenges have been raised by community members in previous consultations over numerous decades, nothing ever changes. It is evident that Aboriginal and Torres Strait Islander people are fed up with being engaged to identify challenges and solutions only to see that the system is never reformed based on their recommendations. The importance of true codesign followed by service improvement initiatives was clear:

*So, the reality for me is, until our own people have a voice and are heard and are listened to, because you can hear people but that doesn't mean you're listening to them, and you actually design things in real codesign with them, we will always be talking about the issue, and I hope something changes in my lifetime.*

*(Aboriginal service provider, Homelessness sector)*

## Discussion

First Nations leaders emphasise that ‘Aboriginal and Torres Strait Islander ways of knowing, being and doing present culturally safe, place-based, and appropriate solutions’ (p.4) (47). Our study brings to light self-determined solutions and drivers of change proposed by Aboriginal and Torres Strait Islander people and service providers in northern Adelaide to address challenges impacting local communities. In considering the evidence presented in this paper, a local Aboriginal leader made a clear statement about the importance of rights and self-determination: “*We are rights-holders, not mere stakeholders*.” She highlighted that the identified drivers of change will begin to dismantle the colonial structures and systems that Aboriginal and Torres Strait Islander communities are forced to live in, yet they represent the beginning of necessary change, not an end point. Through deeply listening to and privileging local voices, we have identified place-based targets for system, service and practice reform. This evidence will inform the development of tailored programs and support advocacy efforts for system-level changes that promote the delivery of health and social services to Aboriginal and Torres Strait Islander people in a more culturally safe and coordinated way.

Time and time again, Aboriginal and Torres Strait Islander people who participated in this study highlighted the importance of culture and place. That is, the need to establish a place for the Aboriginal and Torres Strait Islander community to come together to connect, to practice culture, to feel safe and a sense of belonging: a community hub. The northern region of Adelaide currently has no such place. Targeted programs, infrastructure and opportunities for rebuilding connections across Aboriginal and Torres Strait Islander communities are clearly critical to social and emotional wellbeing and to the remediation of colonial practices of dispossession, exclusion, racism and denial of language and culture. Social resources are protective for Aboriginal and Torres Strait Islander peoples (48) and must be actively and intentionally strengthened in northern Adelaide. Efforts to establish community and cultural hubs and strengthen connections across Aboriginal and Torres Strait Islander communities can be conceptualised in terms of promoting self-determination and building ‘social capital’ - that is, the social relationships and rights of reciprocity of individuals that have been theorised in terms of bonding, bridging and linking factors (49). Whether the western notion of social capital is appropriate from an Aboriginal and Torres Strait Islander perspective is unknown. Inequities in social capital have been identified in Aboriginal communities when operationalised in terms of neighbourhood tenure, ability to get help, feeling valued, perceptions of trust, and being a member of community (6). There are challenges in the evidence for social capital interventions (50) thus implementation of community hubs and evaluations of their impact on Aboriginal and Torres Strait Islander communities and social and emotional wellbeing will require careful deliberation in relation to outcome measures that meaningfully capture community connections, reciprocity, cultural participation and other factors. It is vital that community hubs be established with long-term, significant, and secure investment safeguarded against fluctuating political agendas.

Our study demonstrates that we have a long way to go to establish a service system that is welcoming, culturally safe, and accessible for Aboriginal and Torres Strait Islander people on Kaurna Country, and it calls for multi-level drivers of culturally safe service provision. What is presently in place is clearly insufficient, however the recent launch of the South Australian Aboriginal Health Promotion Plan presents an important progression in local policy (51). This plan specifically focusses on the cultural determinants of health and the associated action plan maps out a pathway towards strengthening culture across the community and service system. Yet a single plan is insufficient and there must be consistent strategic intent throughout the frameworks and action plans that guide program design across health, mental health, family services, community services and other sectors. Furthermore, accreditation standards for health and social services warrant revision to further embed the key features and practices necessary to enact a culturally safe service system for Aboriginal and Torres Strait Islander peoples. Clinical training curricula could also be strengthened to generate emerging cohorts of culturally capable graduates and be reinforced with registration standards and continuous professional development requirements that hold individuals to account in relation to the fundamentals of cultural competence. Mandated annual cultural capability training for frontline workforce is considered central to this agenda.

We heard that for families experiencing poor mental health, homelessness, substance challenges or interactions with the justice system, intensive supports are needed for vulnerable children and young people. These include programs to connect with local Elders and leaders, to spend time On Country, to walk alongside and learn from role models and to be provided vital experiences of connection, pride, hope and opportunity. These findings are not a surprise. Culture and identity are protective factors for various social and health outcomes, including substance misuse (52). Service delivery centred in culture is fundamental to the Aboriginal community controlled primary health care sector in Australia where culture guides and underpins all ways of working (53). Many of the solutions identified in this study echo the characteristics of Aboriginal community controlled models of service delivery relating to community control and participation, self-determination and empowerment, and culturally responsive workforce providing accessible and holistic service delivery (53). What is surprising, though, is that Aboriginal and Torres Strait Islander workforce leading service delivery for Aboriginal and Torres Strait Islander clients has not already been instituted as a core minimal standard of practice across government health and social services. The lack of Aboriginal community-controlled organisations across the health and social service system in northern Adelaide is equally concerning.

Aboriginal community members in this study told of avoiding services for fear of judgement and having children taken away. Temple and colleagues (13) found that experiences of racism leads Aboriginal and Torres Strait Islander Elders to avoid both health and legal services, and Green and colleagues (54) similarly identified troubled interactions between Aboriginal clients and service providers as an inherited legacy of forced removal of Aboriginal children by Australian governments and services. If we are to hope to change the trajectory of Aboriginal and Torres Strait Islander peoples’ lives in northern Adelaide from experiences of racism, discrimination, crisis, shame, isolation, mistrust, and avoidance of services – “*the families with the curtains drawn*”^8^ - to experiences of empowerment, opportunity, stability, trust, connection, access and pride, there must be a major redesign of the way services are provided to community. Participants consistently highlighted the importance of Aboriginal and Torres Strait Islander workforce with lived experience who connect with clients and provide the necessary supports to achieve self-determined goals. Our findings call for investment in both growing and strengthening the Aboriginal and Torres Strait Islander workforce across the service system. This is consistent with the key theme of growing and supporting Aboriginal workforce, identified in recent consultations with Aboriginal communities undertaken in developing the South Australian Aboriginal Health Promotion Plan (51). Our findings suggest that Aboriginal and Torres Strait Islander workforce must lead engagement with community members wherever possible, alongside culturally capable and respectful non-Indigenous workforce. A workforce armed with cultural knowledges, lived experience and clinical qualifications is seen to safeguard cultural safety across services and ensure services are connected to the community and centred in both culture and Western clinical practice models. We know, however, that Aboriginal and Torres Strait Islander workforce that carry this cultural load face multi-level stressors and burdens (55, 56). Therefore extensive supports for workforce must be instituted alongside training, recruitment and retention strategies to counter the challenges of work and community life (57).

Our findings highlight challenges faced by Aboriginal and Torres Strait Islander clients in navigating a fragmented and ‘bewildering’ service system. We heard of the convolutions of red tape, confusing eligibility criteria and transport barriers impacting Aboriginal and Torres Strait Islander clients and instances where services avoided clients because of their complexities. We also heard of significant gaps such as inadequate mental health and drug and alcohol services and rehabilitation programs. The service system must prioritise Aboriginal and Torres Strait Islander clients and be focused on tangible and sustained outcomes that provide benefit in the short term and for generations to come. Our findings call for opportunities for Aboriginal and Torres Strait Islander workforce to build connections with their counterparts in other services, so that they can provide streamlined and timely referrals across services, providing “*warm handovers”* so that clients can connect with and form relationships with trusted workers in partner services and be supported to navigate across the system. Our findings indicate that a network of frontline Aboriginal and Torres Strait Islander workforce across organisations in conjunction with formal inter-organisational partnerships is necessary to promote access and navigation of services and promote strengthened social and emotional wellbeing for Aboriginal and Torres Strait Islander peoples.

There were challenges and tensions raised by some respondents, who were Kaurna people, in relation to the perceived inadequacy of consultation with Traditional Custodians of Kaurna Country about important local matters. There were additional concerns raised in relation to Aboriginal and Torres Strait Islander peoples from other Country holding leadership positions. In a colonised context where Aboriginal and Torres Strait Islander communities are diverse, there are valid questions regarding who represents community. With whom to engage and consult is often uncertain. This is the legacy of intentional displacement practices which saw Aboriginal people moved off their land and separated from Country. In a local context, work is being done to determine appropriate mechanisms for community governance and parliamentary representation. In July 2022 the inaugural Commissioner for First Nations Voice was established in South Australia (58). Our findings speak to the importance of such a role. This appointment followed on from the development of the *Uluru Statement from the Heart* by Aboriginal and Torres Strait Islander people in 2017 (59) that called for a First Nations Voice in Parliament to be enshrined in the Australian Constitution (60). The Commissioner undertook state-wide consultations to determine appropriate mechanisms for electing regional representatives. In March 2023 the South Australian Parliament became the first jurisdiction in Australia to pass a bill for a First Nations Voice. It represents a new chapter in South Australia though the long-term impact and benefit for First Nation’s communities in northern Adelaide are unknown.

Our findings are consistent with a recent study of Aboriginal and Torres Strait Islander youth wellbeing services that welcomed both youth and service provider perspectives and identified the need for safe spaces and points of contact for youth, listening to youth, linking to community members, and early intervention service delivery models (61). The research governance provided by local Aboriginal Elders and service providers was a strength of this study as it promoted trustworthiness through ongoing review and interpretation of emerging findings. The community engagement undertaken by the Senior Engagement and Knowledge Broker who has a long history of living and working in northern Adelaide promoted trust in the research process and connection between the research team and the local community and enabled local perspectives to be brought to light. While we purposively recruited diverse Aboriginal community members, the timing of data collection through COVID-19-related constraints meant that our attempts to recruit the most vulnerable members of the community were unsuccessful. It is a study limitation that our participant sample did not include Aboriginal and Torres Strait Islander participants experiencing great complexity in their lives (e.g., homelessness, substance challenges, and mental health concerns). We did, however, collect shadowed data through the accounts of Aboriginal community members and service providers that support Aboriginal and Torres Strait Islander people in crisis which facilitated inclusion of a comprehensive range of community experiences in the dataset.

Through rapid translation activities, our findings have informed the establishment of the Northern Nungas Network, a collective of frontline Aboriginal and Torres Strait Islander workforce across northern Adelaide who meet monthly. This evidence has also informed advocacy efforts towards a pre-crisis Aboriginal mental health service and an Aboriginal community hub in northern Adelaide. The next phase will see the research team, Aboriginal Governance Panel and project stakeholders working together to co-create a Theory of Systems Change that builds on these findings, models of social and emotional wellbeing (23, 62), and evidence from programs that tackled risks and unmet needs stemming from the social determinants of health to strengthen social and emotional wellbeing in Aboriginal peoples (29, 63–66). In developing this theory of systems change we will collaborate with project partners to explore strategies to promote government accountability, to secure more sustainable and flexible funding, and to identify more effective service commissioning processes. The Aboriginal Governance Panel will guide the research team in determining discrete and feasible community-level, practitioner-level, and service-level implementation projects to developmentally evaluate (67) over the remainder of the research program.

## Conclusion

Aboriginal and Torres Strait Islander communities living on Kaurna Country in northern Adelaide are diverse and demonstrate strength in the presence of challenging conditions. This study documented self-determined solutions proposed by Aboriginal and Torres Strait Islander people and service providers to tackle local challenges, strengthen the service system and promote social and emotional wellbeing. We heard of significant disadvantage, racism and discrimination alongside mental health and substance challenges, financial and housing insecurity, social isolation and extensive multilevel barriers to health and social services access. Participants identified gaps in core services and spoke of services failing to support Aboriginal and Torres Strait Islander people. We identified the key principles, values and ways of working to guide all future service delivery models and highlighted the implementation barriers that must be overcome. Health and social services must carefully consider how they construct culturally safe, strengths-based, tailored and accessible services to promote equity for Aboriginal and Torres Strait Islander peoples. Proactive and pre-crisis service delivery within mental health, drug and alcohol and homelessness services was considered crucial. Aboriginal and Torres Strait Islander workforce leading engagement with clients is seen to safeguard against judgement and discrimination, rebuild community trust in the service system, and promote streamlined access to vital services. Efforts to strengthen community connections and cultural identity through community hubs and cultural events is considered critical. Alongside these place-based efforts considered necessary for systems change, we have highlighted areas where more research is needed to explore how services can be funded and commissioned to strengthen service provision for Aboriginal and Torres Strait Islander people. Future work will see these findings translated into a theory of systems change for northern Adelaide which will represent, from a local First Nations’ perspective, the full gamut of system reforms needed to achieve social and emotional wellbeing in Aboriginal and Torres Strait Islander people.

## Data availability statement

The datasets generated and analysed during this study are not publicly available since data was collected under ethical clearances with strict conditions of confidentiality and protection. Requests to access the datasets should be directed to anna.dawson@sahmri.com.

## Ethics statement

The study was approved by the Aboriginal Health Research Ethics Committee of South Australia (04-20-885) and the Northern Adelaide Local Health Network (HREC 14241). It was conducted in accordance with local legislation and institutional requirements. The participants provided their written informed consent to participate.

## Author contributions

This study was designed by AD, EW, NH, AB, OP, MB, JD, KM, KT, SW, SM, and CA and guided by TS, SC, NR, UL, UW, OB, DB, NC, and AP. Data collection was undertaken by EW and AD. Data analysis was undertaken by EW and AD and refined by the Aboriginal Governance Panel members and research team. Development of the manuscript was led by AD and EW alongside NH, TB, KL, and CH. All authors contributed to the article and approved the submitted version.

## Funding

The project is funded by the National Health and Medical Research Council (NHMRC #1165364). AB is funded by a National Health and Medical Research Council Senior Research Fellowship (NHMRC#1137563).

## Conflict of interest

The authors declare that the research was conducted in the absence of any commercial or financial relationships that could be construed as a potential conflict of interest.

## Publisher’s note

All claims expressed in this article are solely those of the authors and do not necessarily represent those of their affiliated organizations, or those of the publisher, the editors and the reviewers. Any product that may be evaluated in this article, or claim that may be made by its manufacturer, is not guaranteed or endorsed by the publisher.

## Footnotes

^1^The Kaurna people are the traditional custodians and first inhabitants of the region known, following colonisation, as the Adelaide Plains.

^2^*Taingiwilta Pirku Kawantila* is the project name given by Kaurna language and knowledge holders - Kaurna Warra Karrpanthi - meaning ‘Strong Community in the North’.

^3^*Wardliparingga* means ‘house river place’ in Kaurna language and is also a story from the dreaming which reflects the Milky Way in the Karrawirraparri River which runs alongside the South Australian Health and Medical Research Institute. This name was given by Kaurna language and knowledge holders, Kaurna Warra Karrpanthi.

^4^The Stolen Generations is the collective name given to the Aboriginal and Torres Strait Islander peoples who, as children in the 20^th^ Century, were forcibly removed from their families under successive government policies (Human Rights and Equal Opportunity Commission, 1997).

^5^Aboriginal community controlled organisations are unique in that they are governed by a Board of Management comprised of Aboriginal community members.

^6^Children under Guardianship Orders are under the custody or guardianship of the Chief Executive Officer of the South Australian Department of Child Protection after being removed from their carers due to risk of serious harm.

^7^‘Nunga’ is the terminology used for self-identification by Aboriginal people in South Australia.

^8^This is a phrase used by a study Chief Investigator to describe the families that have had negative experiences with services, who avoid services and are socially isolated in community.
